# Inequalities and Trends in Under-Five Mortality Between Formal and Informal Areas in Ouagadougou, Burkina Faso

**DOI:** 10.1007/s11524-024-00878-4

**Published:** 2024-08-30

**Authors:** Bruno Yempabou Lankoandé, Yacouba Compaoré, Kassoum Dianou, Souleymane Sanogo, Abdramane Soura, Abdoulaye Maïga, Agbessi Amouzou

**Affiliations:** 1 Institut Supérieur des Sciences de la Population (ISSP), Université Joseph Ki-Zerbo, Ouagadougou, Burkina Faso; 2 Centre de Recherche en Démographie, Université Catholique de Louvain, Louvain‐la‐Neuve, Belgium; 3 Institut de Démographie et de Socioéconomie, Université de Genève, Geneva, Switzerland; 4 Department of International Health, Johns Hopkins Bloomberg School of Public Health, Baltimore, MD USA

**Keywords:** Under-five mortality, Cause-specific mortality, Ouagadougou Health and Demographic Surveillance System, Free health policy, Informal areas

## Abstract

The physical expansion of the city of Ouagadougou, the capital and largest city of Burkina Faso, subsided in 2015 after the government banned land speculation that contributed to the growth and entrenchment of informal areas. The government subsequently implemented social policies such as free health care for pregnant women and children under 5 years of age. Against this background, we tested the convergence of under-5 mortality trends between formal and informal areas in the city between 2010 and 2019; data covering that period came from the Ouagadougou Health and Demographic Surveillance System (HDSS). The analyses included the calculation of all-cause and cause-specific mortality rates, the implementation of a Poisson regression model, and competing risk models. Over the study period, children in formal areas had lower mortality than those in informal areas. However, the inequality gap decreased over time due to a faster mortality decline in informal areas. This decline was explained by a rapid decline in deaths from malaria and other causes including sepsis, HIV/AIDS, measles, meningitis, and encephalitis. The pursuit of upgrading informal areas and the implementation of social policies targeting the poorest are likely to accelerate the mortality decline in Ouagadougou overall.

## Introduction

The urban population is growing rapidly in sub-Saharan Africa (SSA). In 2020, 44% of the inhabitants of this region lived in cities, and this proportion is expected to exceed 50% in 2040 according to United Nations projections [1]. This urbanization is occurring in part due to significant natural growth of country populations due to high fertility and declining mortality [2, 3] and has been accelerated by rural–urban migration of young adults in search of better living conditions. In addition, many African cities are rapidly expanding in geographical size, including as villages located on the outskirts are reclassified over time as urban areas and thus considered within the boundaries of the city [4].

In a context of slow and limited economic development, this sustained growth of the urban population in SSA is driven primarily by settlement in informal areas, which are typically inhabited by poor populations in search of low-cost housing [5–7]. These areas, also called “slums,” “irregular neighborhoods,” and “underdeveloped areas,” are characterized by a lack of access to basic social services such as drinking water and sanitation and a very limited health care supply. Although the configuration of these areas and the living conditions of their inhabitants vary from city to city, it is established that health indicators across their populations are poorer compared with populations in formal areas. Various studies have highlighted this gap, which is especially pronounced regarding children because their health status remains highly dependent on the health environment and access to health care services. For example, in Ouagadougou, Burkina Faso, studies have shown higher under-five mortality rates in informal areas compared with formal ones. This health disadvantage is observed throughout the neonatal, infant, and juvenile period [8, 9]. Results from similar studies conducted in Kenya also found higher mortality among children in informal areas compared with children living in other urban areas as well as children living in rural areas [10, 11]. Besides mortality, the health disadvantage of informal areas is observed for other health outcomes such as malnutrition, diarrhea, and respiratory infections [12, 13].

Several initiatives with the goal of improving living conditions in informal areas have been implemented with support from international organizations such as the World Bank and the United Nations Human Settlements Programme (UN-Habitat). They range from the simple relocation of people from informal areas to formal areas, to policies on upgrading and restructuring informal areas in collaboration with local authorities and non-governmental organizations (NGOs) [14]. In recent years, most projects have focused on improving conditions in informal areas in line with Goal 11 of the Sustainable Development Goals (SDGs), which aims to ensure access for all to decent housing and basic social services. The basis of this upgrading strategy is land security via legal recognition by the relevant authorities of property held by residents to prevent eviction. This step is intended to boost and promote residents’ incentive to invest more in their housing. At the same time, the provision of basic social services such as drinking water, sanitation, and the construction of adequate roads and sanitary infrastructure—all important components of most upgrading approaches—makes it possible to better integrate informal areas into a city. Examples include slum upgrading projects in Burkina Faso, Ghana, Kenya, and Zimbabwe [14–16].

By helping to reduce the health disadvantage associated with living in informal areas, the slum upgrading strategy is expected to accelerate the bridging of gaps in the health status of populations living in both types of areas. This hypothesis was based on a theoretical framework stating that the implementation of adequate public policies can lead to a convergence of health status between elite and non-elite populations [17].

The objective of this paper is to analyze trends and inequalities in under-five mortality between formal and informal areas in Ouagadougou, the capital of Burkina Faso. More specifically, it aims to (i) depict the inequalities in all-causes mortality and cause-specific mortality according to the type of area and (ii) review the effects of different urban planning and health policies on the differential of under-5 mortality between formal and informal areas. Even though the disadvantages experienced by children living in informal areas have been well documented in SSA and elsewhere, analysis of the effects of urban planning and health policies on changes in health inequality has rarely been conducted in Burkina Faso.

## Context

As is the case in most other cities in SSA (and especially capital cities), the population of Ouagadougou has grown considerably in recent decades. It increased fivefold between 1960 and 2020 to reach 2.5 million inhabitants, which represents nearly half (45.1%) of the country’s urban population [18]. Meanwhile, the land size of the city grew at a similar rate from 1960 to 2014, increasing from 6000 hectares (23 square miles) to 30,250 hectares (about 117 square miles) [19].

The urban development of Ouagadougou stems from trends established during the colonization period, with formal areas occupied by Europeans and the indigenous population living in informal areas. The housing situation and policies of these informal areas have evolved over time, sometimes in response to proposals and support from international organizations. National policy changes in 1987 formalized the right to private property and the control of the customary chiefdom over land, particularly in villages on the outskirts of towns and cities [20]. These changes fueled land speculation that in many cases resulted in the erection of informal areas on the periphery pending formal allotment operations. Following allotment, these lands turned into formal areas, became more valuable over time and were subsequently sold, thus expanding the boundaries of the city; meanwhile, other informal areas continued to be created and the cycle repeated itself. This mode of subdivision led to various malpractices and conflicts that precipitated a suspension of the practice in 2015 in the country’s two main urban areas, Ouagadougou and Bobo-Dioulasso.

The new strategy was to focus on improving living conditions in informal areas through the implementation of various projects. This had already been underway in some places. For instance, the project for the development and opening up of peripheral districts (PADQP), whose activities covered the period 2007–2011, aimed to facilitate access to basic social services for populations living in informal areas [14]. More specifically, a partnership between private operators and the National Office of Water and Sanitation (ONEA) has made it possible to test a drinking water supply system in the homes of people living in informal areas. Two districts within the Ouagadougou Health and Demographic Surveillance System (HDSS) benefited from this project (Nonghin and Polesgo). Similarly, the project “Sustainable sanitation in the informal and peripheral districts of Ouagadougou in Burkina Faso” (PERISAN) resulted in the provision of 15,913 toilets for the benefit of 80,406 people in 30 informal areas, including the Ouagadougou HDSS, over the period 2014 to 2017 [21].

The city has also benefited from the Ouagadougou Peripheral District Sanitation Project (SPAQPO), which aimed to improve rainwater drainage systems, collect and recover solid waste and open up peripheral districts. Most recently, the Ministry of Health and Public Hygiene in collaboration with international partners launched a project to restructure informal areas in several regions of the country, including Ouagadougou. Key elements of the initiative include developing and improving roads and establishing basic social infrastructure. The pilot phase of this project is currently being implemented in a rural municipality (Saaba) at the periphery of Ouagadougou.

This non-exhaustive list of projects carried out in informal areas is likely to improve the health conditions of the populations living there and consequently contribute to accelerating convergence of mortality risk.

## Data and Methods

### Data

**Fig. 1:**
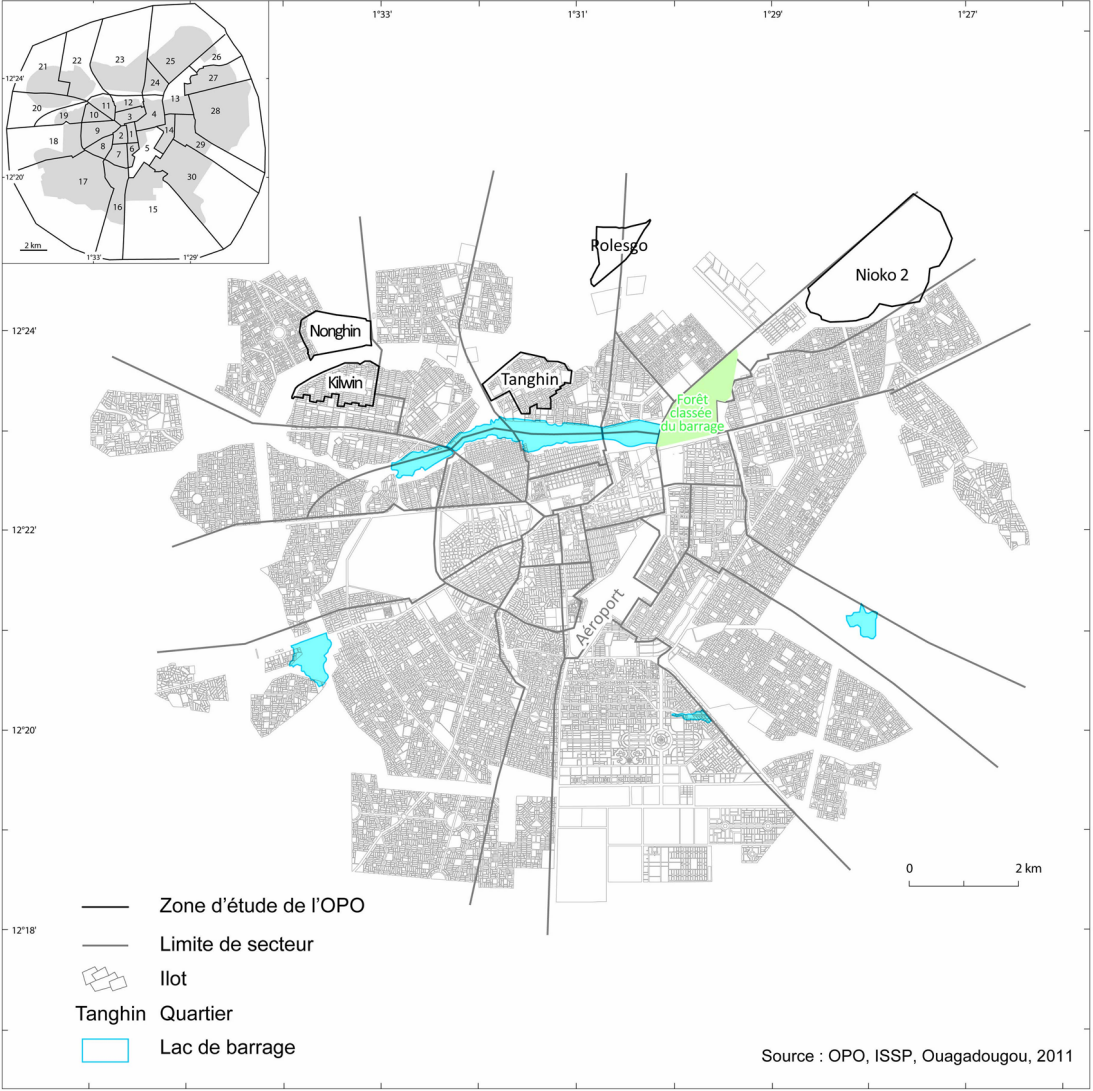
Districts included in Ouagadougou HDSS (areas under demographic surveillance)

The data for this paper’s analysis come from the Ouagadougou HDSS, one of two urban HDSS in SSA [22, 23]. Established in 2008, the HDSS includes five districts located on the northern outskirts of the city of Ouagadougou. (Fig. 1). These five districts were divided into two formal areas (Kilwin, Tangin) and three informal areas (Polesgo, Nioko 2, Nongin) [24], with total populations under surveillance estimated at 32,399 and 58,268 inhabitants, respectively, in 2019.

Data in the HDSS are collected on the entire population and updated through annual regular rounds of visits. They contain information on vital events (births, migrations, deaths, etc.) and demographic and socioeconomic characteristics (level of education, marital status, religion, ethnicity, occupation status, household assets, etc.). In the event of death, probable causes of death are collected through the verbal autopsy method and analyzed using Inter-VA software [25]. The derived causes of death are then classified according to the International Classification of Diseases (ICD) version 10 (ICD-10) [26].

The data considered in this analysis cover the period 2010–2019. The contribution of children under 5 years of age in terms of person-years over the period considered is 133,257.3. During those 10 years, there were a total of 814 deaths in that age group. Among these deaths, probable cause of death was estimated for 71% of cases. The cause of death was determined for 67% and 73%, respectively, in formal and informal areas. The inability to ascertain causes of death for many of the children can be explained by refusals, resettlement in other parts of the city, the absence of the ideal respondent, and the lack of information on some verbal autopsy questionnaires to determine the cause of death. For this analysis, causes of death were grouped into six groups: malaria; respiratory diseases; neonatal causes (congenital malformation, prematurity, birth asphyxia, neonatal pneumonia, neonatal sepsis and other unspecified cause of death); diarrhea; malnutrition; and other causes of death (e.g., non-obstetric sepsis, HIV, measles, meningitis, and encephalitis).

### Statistical Analyses

First, we conducted an analysis of all-causes mortality trends by type of area based on the calculation of annual mortality rates for children under 5 years of age. These rates were obtained by dividing the number of deaths by the number of person-years lived.

In a second step, we smoothed observed mortality trends in each type of area to eliminate random fluctuations and highlight inequalities in mortality over time. This smoothing was done using Poisson modelling from a person-periods file [27]. The dependent variable was the number of deaths with time as the main independent variable. For the smoothing, this independent variable was decomposed using a cubic spline interpolation [28].

The cause-specific mortality rates were calculated by period for each type of area to overcome issues of sample size. The analysis period (2010–2019) was divided into two sub-periods: 2010–2015 and 2016–2019. The first sub-period corresponds to the beginning of demographic surveillance excluding the baseline year (2009) and the second sub-period coincides with the implementation of the policy of free health care for pregnant women and children and the cessation of land subdivision operations. In this analysis, we redistributed deaths with undetermined cause of death proportionally to cases with determined cause of death. Finally, we implemented a competing risk model for each group of cause of death with sub-period and type of area as independent variables and an interaction term between the two. The objective of this modelling was to capture over time the contribution of each group of causes to the gap in under-5 mortality between formal and informal areas. The competing risk model takes into account the fact that children are exposed at the same time to different causes of deaths that compete [29].

To test the hypothesis of mortality convergence between formal and informal areas over the 10-year analysis period, we used a Poisson regression model using the sub-periods, type of area, and interaction between these two variables as the main explanatory variables. The objective was to assess the trends in the mortality gap over time. The control variables included the child’s sex, the characteristics of the mother (educational level, age group, ethnic group, religion, and marital status), and the characteristics of the household (access to drinking water, type of toilet, and household size). The distribution of these characteristics by type of area and by sub-period is presented in the appendix (Table 4).

## Results

**Fig. 2:**
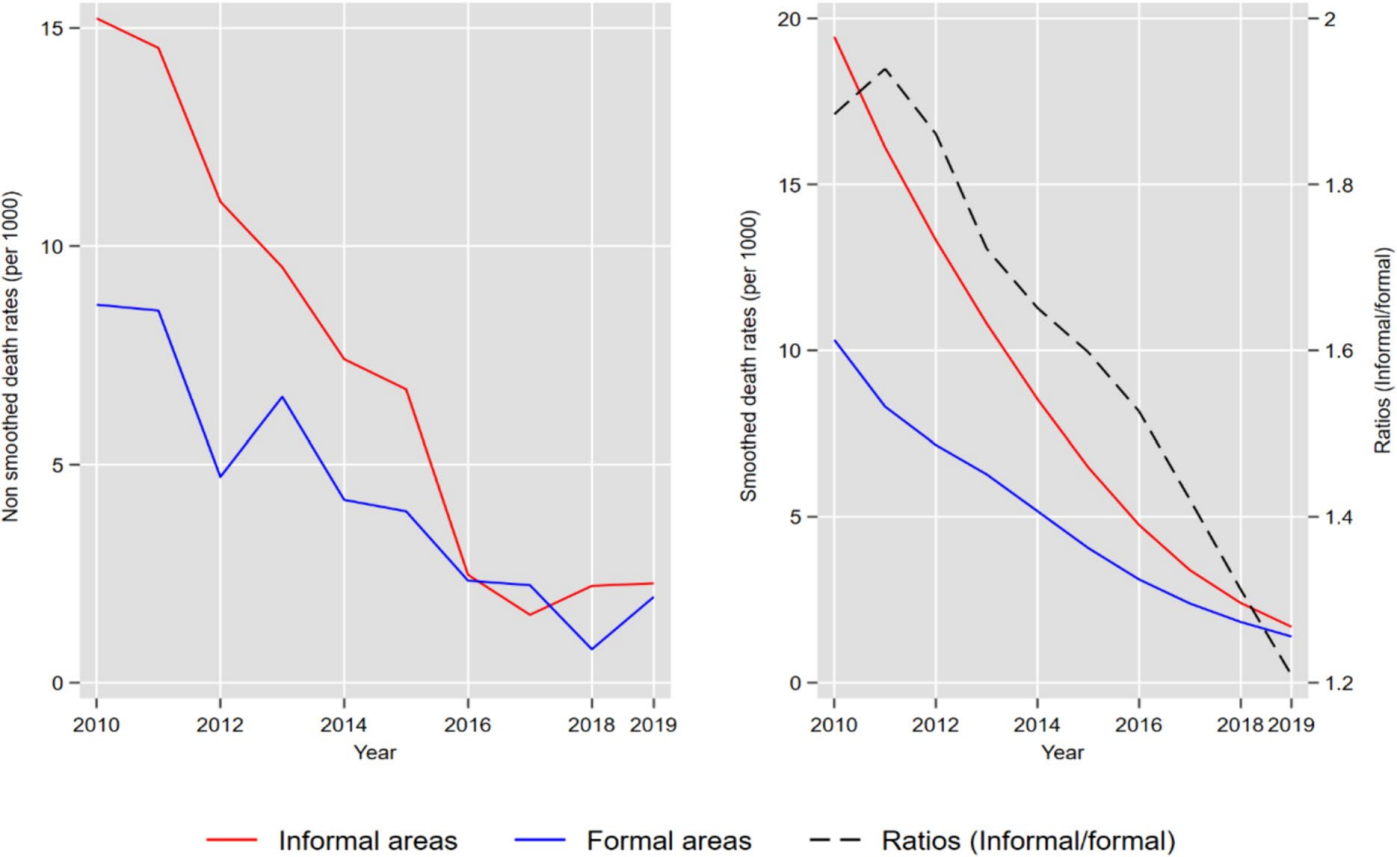
Trends in under-5 mortality rate by type of area, 2010–2019

Figure 2 presents the trends in the under-5 mortality rate by type of area over the period 2010–2019. The first graph shows the non-smoothed mortality rates as computed from the data, while the second graph shows the smoothed rates. In addition to the graph, Table 1 quantifies the absolute and relative differences in mortality levels between formal and informal areas by sub-period.

**Table 1:** Under-5 mortality rates (per 1000) by period and type of area, 2010–2019

Period	Formal	Informal	Absolute difference	Relative difference
2010–2015	6.2 [5.3–7.2]	10.45 [9.6–11.4]	4.3	69.6
2016–2019	1.8 [1.3–2.7]	2.1 [1.7–2.6]	0.3	16.3
Total	4.6 [4.0–5.3]	6.9 [6.3–7.4]	2.3	48.9

The relative difference is in percentage

Over the entire period 2010–2019, changes in mortality levels show that children from informal areas were at a higher risk of death than children living in formal areas. However, Fig. 2 suggests a reduction in the gap in under-5 mortality between formal and informal areas over time, although the informal areas remained disadvantaged in 2019. This reduction in the mortality gap was driven mainly by a faster decline in under-5 mortality in informal areas compared with formal areas. The mortality ratio between informal and formal areas declined from 1.9 in 2010 to almost 1.2 in 2019. Similarly, the absolute difference in mortality levels decreased from 4.3 per 1000 over the 2010–2016 sub-period to 0.3 in the 2016–2019 sub-period. As for the relative difference, it decreased from 69.6 to 16.3% from the first to the second sub-period.

**Fig. 3:**
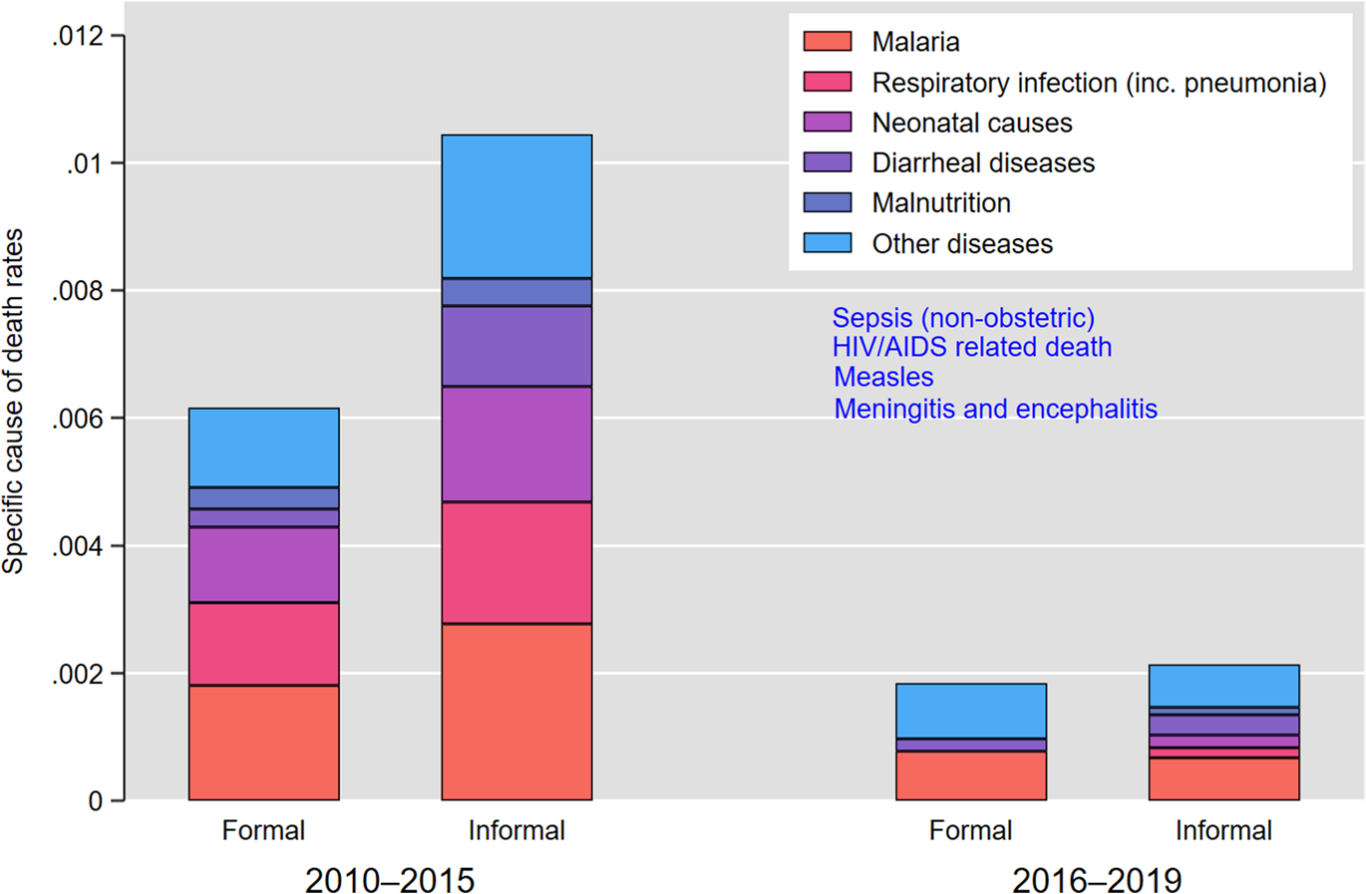
Cause-specific under-5 mortality rate by type of area and period, 2010–2019

Figure 3 presents cause-specific mortality rates for the two sub-periods. Malaria, diarrhea, and neonatal causes were the main causes of death regardless of the area and observation period. Mortality due to respiratory infections was particularly salient during the first sub-period in both areas. Some causes were not observed during the second sub-period in formal areas due to issues of sample size.

The observed decline in the gap of all-causes mortality between formal and informal areas was mainly related to the decline observed in mortality from malaria and the other causes of deaths. First, Table 2 shows that children living in informal areas were more likely to die from malaria and from other causes compared with those living in formal areas. The sub-distribution hazard ratio is respectively estimated at 1.7 (95% CI: 1.13–2.53) and 1.4 (95% CI: 1.09–1.83) for deaths due to malaria and the other causes. Second, as shown by the interaction term, the disadvantage of informal areas has reduced over time, with a sub-distribution hazard ratio of 0.44 (95% CI: 0.17–1.14) for mortality due to malaria and 0.59 (95% CI: 0.32–1.07) for mortality due to the other causes. These results are, however, only marginally significant at *p*-value < 10%.

**Table 2:** Sub-distribution hazard ratios for under-5 mortality due to malaria, diarrhea, and other causes, 2010–2019

	Malaria			Diarrheal diseases			Other causes		
	SHR	*P*-value	95% CI	SHR	*P*-value	95% CI	SHR	*P*-value	95% CI
Type of area									
Formal	1			1			1		
Informal	1.69	0.011	1.13–2.53	4.77	0.001	1.89–12.0	1.42	0.007	1.09–1.83
Period									
2010–2015	1			1			1		
2016–2019	0.48	0.061	0.22–1.03	0.76	0.738	0.15–3.89	0.45	0.002	0.27–0.74
Type of area/period									
Informal 2010–2015	1			1			1		
Informal 2016–2019	0.44	0.091	0.17–1.14	0.33	0.23	0.22–2.01	0.59	0.084	0.32–1.07
Number of deaths	153			61			364		

*SHR*, sub-distribution hazard; *CI*, confidence interval

Table 3 presents the relative incidence of under-5 mortality by type of area, sub-period, interaction between type of area and sub-period, and other control variables. First, it appears that mortality declined over the full 10-year analysis period. As expected, mortality among children living in formal areas was lower compared with those in informal areas. The mortality inequality remained after controlling for the sex of the child, household size, water source, type of toilet used, level of education, age group, ethnicity, religion, and marital status of the mother. All things being equal, the risk of death for children under 5 years of age was 60% higher in informal areas compared with formal areas over the analysis period. However, this net disadvantage of informal areas was not constant. It declined over time with a clear and statistically significant difference at *p*-value < 10% between the two sub-periods.

**Table 3:** Relative incidence of under-5 mortality in the Ouagadougou HDSS, 2010–2019

Variables	IRR (raw effects)	*P*-value	95% CI	IRR (net effects)	*P*-value	95% CI
Area type						
Formal	1			1		
Informal	1.6	0.000	1.28–1.97	1.50	0.002	1.16–1.94
Sub-periods						
2010–2015	1			1		
2016–2019	0.33	0.000	0.20–0.55	0.34	0.000	0.21–0.57
Area type*sub-period						
Informal 2010–2015	1			1		
Informal 2016–2019	0.59	0.069	0.33–1.04	0.58	0.062	0.32–1.03
Gender						
Male				1		
Female				1.11	0.225	0.94–1.31
Level of education of the mother						
None				1		
Primary				1.02	0.87	0.82–1.26
Secondary and above				0.78	0.092	0.58–1.04
Not specified				0.91	0.612	0.64–1.30
Age group of the mother						
Under 35 years of age				1		
35 years and over				1.25	0.088	0.97–1.62
Ethnicity of the mother						
Mossi				1		
Others				1.15	0.309	0.88–1.52
Religion of the mother						
Catholic				1		
Not Catholic				0.91	0.318	0.77–1.09
Marital status of the mother						
Married				1		
Unmarried				0.81	0.435	0.47–1.38
Household water source						
Other sources (wells, hand pumps, street vendors, rainwater)				1		
Public fountain				0.97	0.755	0.78–1.20
Valve				0.74	0.089	0.52–1.05
Type of toilet in the household						
No toilet				1		
Simple latrines				0.68	0.091	0.43–1.06
Other (flushing and ventilated latrine)				0.97	0.906	0.57–1.64
Household size						
Less than 5 people				1		
5 people or more				1.11	0.247	0.93–1.34

*IRR*, incidence-rate ratios

The analysis also revealed that factors such as the age of the mother, the source of water used, type of toilet used, and level of education were associated with under-5 mortality, although these results were only significant at the *p*-value < 10% threshold. In terms of maternal age, children with older mothers (aged 35 years or older) were at higher risk of mortality than children of younger mothers. The availability of a tap in the household was associated with lower child mortality compared with the use of a public fountain or other water sources.

## Discussion

This research focused on inequality trends in under-5 mortality between formal and informal areas in Ouagadougou, the capital and largest city of Burkina Faso. The analysis was based on longitudinal data collected in the Ouagadougou HDSS over a recent period: 2010–2019. Distinct from most other previous research on the topic, this analysis assessed the association between the changes in child mortality gap between the two types of areas with contextual factors and various national and local health and urban development policies affecting the city of Ouagadougou. Access to reliable data on cause-specific mortality, which are rarely available at the community level in Burkina Faso, made it possible to explain mortality trends.

The analysis confirmed the existence of higher levels of mortality among children residing in informal areas compared with those in formal areas as documented elsewhere in the literature [8, 12]. This greater vulnerability of children living in informal areas is mainly due to socio-demographic, economic, and behavioral factors [30–32]. However, mortality differentials have narrowed over time due to a faster decline in mortality in informal areas. The main source of this reduction was a sharp decline in deaths from malaria and other causes (e.g., sepsis, HIV/AIDS, measles, meningitis, and encephalitis) in informal areas. Overall, these results give credit to the hypothesis that mortality gaps can be narrowed significantly through the combination of rapid improvement in living conditions and better access to and use of health care services in informal areas. The improvement of living conditions in informal areas may be associated with the ban on formal allotment operations and projects that increased access to services in those areas and promoted restructuring of them. The reduction in malaria and infection-specific deaths may be related to improved care seeking following the Burkinabe government’s introduction, in March 2016, of a policy of free basic health care for pregnant women and children [33].

The quest for residential autonomy is one of the reasons that people settle in informal areas in the city of Ouagadougou. People often stay in these areas in the hope that the government will undertake subdivision operations that would allow them to become the formal owners of the occupied lands. This approach makes sense to them on many levels because the land usually gains value in the event of a subdivision. However, it also means that most residents of informal areas have little or no incentive to invest more in their housing, which is usually demolished during the process of formal allocation of plots. The benefits of this land occupation strategy have been severely challenged and constrained in recent years after the government in 2015 halted subdivision operations in the city of Ouagadougou. Yet, on a positive level, the measure may encourage people to invest more in their living environment, and this in turn may have a beneficial impact on their health and, consequently, on the mortality of children living in informal areas. This is the case, for example, of the Polesgo district, where communities sometimes organized in association, with support from some NGOs, to improve their living environment through the installation of public fountains and the construction of social infrastructure such as health centers and schools [14].

Finally, results from the analysis underscore that some public policies are more likely to benefit the poorest people than wealthier ones. Other recent work showed that the policy of free health care for pregnant women and children implemented since March 2016 has reduced inequalities in access to care between the poor and the rich in Burkina Faso [33]. Similar results have been documented in Sierra Leone [34]. In addition, some initiatives in Burkina Faso are more beneficial for the poorest because they specifically target the poor. This is the case of social safety nets (including those with cash transfer components) and certain projects such as the solidarity approach project in reproductive health, known as PASSAGE. This project, implemented between 2006 and 2009, aimed to improve services provision, access, and quality of care for certain target populations such as adolescents, pregnant women and their families, and the most deprived people and communities, as identified by NGOs and local associations [35].

This study has some limitations. Firstly, the Ouagadougou HDSS is not representative of the city of Ouagadougou as a whole. However, the longitudinal nature of the data makes it possible to highlight causal mechanisms that would not be different for the rest of the city. Secondly, it was difficult to establish through a statistical analysis a causal relationship between policies, projects, and changes in mortality inequality between formal and informal areas over the analysis period.

Beyond these limitations, this paper contributes to evidence on socioeconomic inequalities in health in cities in low- and middle-income countries. On the one hand, the health disadvantage of the urban poor persists in most African cities and seems to contribute precisely to the erosion of the urban health advantage compared to rural areas [36]. On the other hand, the example of cities such as Ouagadougou suggests a rapid decline in recent years in mortality that has been particularly steep in informal areas and thus has resulted in a growing convergence of mortality levels between informal and formal areas. The implementation of adequate urban policies for the benefit of the poorest could further accelerate the erasure of the mortality gap in cities in low- and middle-income countries and therefore contribute to the achievement of SDG target 3.2 to reduce preventable deaths among newborns and children under age 5 in general.

## Data Availability

Data used in this study come from routine data collected in the Ouagadougou Health and Demographic Surveillance System (HDSS). Data are available upon requested.

## Appendix

**Table 4:** Distribution (%) of socioeconomic characteristics by type of area and period, 2010–2019

	2010–2015 sub-period		2016–2019 sub-period		2010–2019 overall period	
	Formal	Informal	Formal	Informal	Formal	Informal
Gender						
Men	54.6	50.3	56.1	51.2	55.2	50.7
Women	45.4	49.7	43.9	48.8	44.8	49.3
Education						
None	52.4	61.0	50.9	58.0	51.8	59.6
Primary	20.4	20.4	21.5	21.8	20.8	21.1
Secondary	18.4	10.6	21.0	12.9	19.4	11.7
Unknown	8.9	8.0	6.6	7.3	8.0	7.6
Age group						
< 35 years	86.5	91.9	85.0	90.9	85.9	91.4
35 years and older	13.5	8.1	15.0	9.1	14.1	8.6
Ethnic group						
Mossi	88.8	92.0	89.3	92.0	89.0	92.0
Others	11.2	8.0	10.7	8.0	11.0	8.0
Religion						
Muslim	61.5	61.8	59.9	58.5	60.9	60.3
Others	38.5	38.2	40.1	41.5	39.1	39.7
Marital status						
Married	87.1	93.7	88.5	95.1	87.6	94.4
Unmarried	12.9	6.3	11.5	4.9	12.4	5.6
Household size						
< 5 persons	11.9	29.9	11.4	24.2	11.7	27.3
5 persons or more	88.1	70.1	88.6	75.8	88.3	72.7
Household water source						
Public fountain	39.4	71.4	23.3	75.7	33.2	73.4
Valve	51.7	3.5	71.4	9.9	59.3	6.5
Other sources (wells, hand pumps, street vendors, rainwater)	8.9	25.1	5.4	14.4	7.5	20.1
Type of toilet in the household						
Simple latrines	82.6	90.4	68.8	87.0	77.3	88.8
No toilet	0.3	3.3	0.2	0.7	0.2	2.1
Other (flushing and ventilated latrine)	17.1	6.3	31.0	12.4	22.5	9.1
Total	100.0	100.0	100.0	100.0	100.0	100.0
